# Development of a novel method for identification and quantification of birch pollen allergens in parallel

**DOI:** 10.1186/2045-7022-4-S2-P8

**Published:** 2014-03-17

**Authors:** Jelena Spiric, Anna Maria Engin, Michael Karas, Stefan Vieths, Andreas Reuter

**Affiliations:** 1 Paul-Ehrlich-Institut, Divison of Allergology, Langen, Germany; 2 Goethe-Universität Frankfurt am Main, Institute of Pharmaceutical Chemistry, Frankfurt, Germany

## Background

Determining the content of individual allergens at isoform level has become important for comprehensive standardization and quality control of allergen products as the content of individual allergens has an impact on the potency of allergen extracts. Mass spectrometry (MS) is a dominant and indispensable proteomic tool that has been employed for allergen analysis. Therefore, the main focus of this study was to develop a MS method capable of identifying peptides for quantification of individual Bet v 1 isoforms, and to quantify the entire protein profile including allergenic and non-allergenic proteins of birch pollen in a single run.

## Methods

Identity of Bet v 1 isoforms in the extract was determined using 2D-PAGE coupled with MS. An analytical liquid chromatography (LC) -ESI-MS^E^ method was applied for recording protein profile, and quantifying allergenic and non-allergenic proteins in birch pollen.

## Results

2D-PAGE analysis revealed the presence of eighteen different, Bet v 1, isoforms in the extract. Four Bet v 1 isoforms have an isoform-specific peptide, allowing individual and unambiguous quantification. There was not a single peptide shared by all Bet v 1 isoforms. Furthermore, fifty percent of the Bet v 1 isoforms identified with 2D-PAGE /MS were detected in solution as well. All five officially accepted birch pollen allergens and several non-allergenic proteins were quantified using a single calibrant, with Bet v 1 being the most abundant protein.

## Conclusion

Our study indicates that the isoform pattern of Bet v 1 is more complex than previously described, and it does not allow individual quantification of all Bet v 1 isoforms by MS. However, a combination of three peptides can be used for the quantification of Bet v 1 in total. Additionally, the method grants quantitative evaluation of the protein profile. This proteomic approach can further be used for quantitative analysis of isoallergens of Bet v 1 with different clinical relevance since several tryptic peptides contain amino acids residues indicative for isoallergens with high, medium and low IgE binding capacity.

